# COVID-19-Pandemie und politische Institutionen: Lokale Politik und Wahlen

**DOI:** 10.1007/s11615-021-00302-5

**Published:** 2021-01-29

**Authors:** Norbert Kersting

**Affiliations:** Institut für Politikwissenschaft, Universität Münster, 48151 Münster, NRW Deutschland

**Keywords:** Corona-Pandemie, Wahlkampf, Lokalpolitik, Kommunalwahl, Briefwahl, Corona-Pandemic, Electoral campaigning, Local politics, Local election, Postal voting

## Abstract

Die Covid-19-Pandemie führte zu massiven Einschränkungen und Beeinträchtigung der politischen Öffentlichkeit. Die Befragung von über 1500 Ratsmitgliedern in Nordrhein-Westfalen zeigte, dass auf lokaler Ebene Gemeinderäte nur eingeschränkt arbeiten konnten. Nach einem vollständigen Lockdown im März 2020 übernahmen häufig kommunale Haupt- und Finanzausschüsse die Arbeit der Gemeinderäte. In einigen Kommunen tagten die Räte in halber Besetzung. In dieser „Stunde der Exekutive“ konnten viele Kommissionen und Ausschüsse, aber auch Veranstaltungen von zivilgesellschaftlichen Organisationen gar nicht stattfinden. Im Vorfeld der Kommunalwahlen im September 2020 kam es zu Problemen bei der Registrierung von Parteien. Es mangelte insbesondere an räumlicher wie auch digitaler Infrastruktur. Mit angepassten und abgemilderten Corona-Schutzverordnungen sowie besonderen Regelungen im Rahmen der Kommunalwahl in Nordrhein-Westfalen wurde der Prozess der Registrierung etwas erleichtert und die Fristen um wenige Tage erweitert. Im Wahlkampf hatten insbesondere neue, kleinere Parteien sowie neue Kandidaten Probleme sich darzustellen und somit häufig Nachteile gegenüber den Amtsinhabern. Straßen-Wahlkampf, Podiumsdiskussionen und Tür-zu-Tür-Kampagnen fanden nur sehr begrenzt statt. Im Rahmen der „Zwangsdigitalisierung“ lag insbesondere bei den etablierten Parteien erstmalig ein deutlicher Schwerpunkt auf den digitalen Kanälen. Ein Verschieben der Wahl wurde vor allem durch die Ratsmitglieder der Kleinparteien eingefordert. Eine vollständige Briefwahl wurde mehrheitlich in allen Parteien abgelehnt.

## Abstract (en)

The COVID-19 pandemic has caused massive restrictions on public life. A survey of more than 1500 local councilors showed that councils at the local level are restricted in their capacity. After a total lockdown in March 2020 at the local level, the local central committee (Hauptausschuss) took over, substituting for and representing the full city council’s local decision-making capacity. Later on, in some cities, councils were able to implement council meetings with half of the councilors. In this “executive hour,” several local committees and commissions as well as political events organized by the civil society could not be implemented.

Before the local elections on September 22 in North Rhine–Westphalia, the pandemic affected the registration of political parties, which became problematic. Appropriate space, as well as digital infrastructure, was lacking. With more mitigated COVID-19 restrictions and new regulations for local elections by the province of North Rhine–Westphalia, electoral party registration was facilitated, and deadlines were extended. In particular, smaller political parties and new candidates for the mayoral direct election experienced problems in presenting themselves during electoral campaigns; new parties and candidates were often disadvantaged compared with the political incumbent and the established political parties.

Furthermore, electoral street campaigning, panel discussions, and canvassing were extremely limited. In this time of “forced digitization,” the established political parties in particular were able to focus on e‑campaigning. Because of these disadvantages, smaller parties requested postponement of the local elections. The majority in all political parties refused postal voting.

## Einleitung

Die COVID-19-Pandemie führte mit ihren restriktiven Abstandsregelungen zur Selbst-Isolation und durch den Lockdown zu einem Abbau von sozialer und politischer Öffentlichkeit. Dabei war sie auch – aufgrund der hiermit verbundenen Zwangsdigitalisierung – ein Stresstest für die digitale Infrastruktur und vorhandene Software. Hier wurden Defizite sowohl in der Online- als auch in der Offline-Kommunikation offensichtlich. Diese demokratischen Defizite betrafen auf kommunaler Ebene die repräsentativen Institutionen für politische Entscheidungsprozesse, d. h. das Tagesgeschäft der Gemeinderäte wie Gemeinderats- und Ausschusssitzungen ebenso wie die Umsetzung von Wahlen. Wahlen und Abstimmungen sind in Friedenszeiten selten durch externe Faktoren beeinflusst worden. Zwar zeigte sich durch Umweltkatastrophen, wie z. B. die Fukushima-Reaktor-Explosion, ein unterschiedliches politisches Wahlverhalten. Die Umsetzung der Wahl war hierdurch allerdings nicht grundlegend beeinträchtigt (Norris 2012, 2014).

Nach dem Ausbruch der COVID-19-Pandemie Ende Dezember 2019 in der chinesischen Stadt Wuhan breitete sich die Pandemie weltweit rasant aus (Bundesministerium für Gesundheit 2020). Im Januar 2020 wurden erste Fälle von infizierten Personen in Deutschland offensichtlich. Anfang März wurde deutlich, dass die Epidemie Deutschland erreicht hatte. Erste Maßnahmen zur Begrenzung und zur Eindämmung, wie z. B. die Risikobewertung von Großveranstaltung wurden getroffen. Mitte März 2020 wurden z. B. in Nordrhein-Westfalen Kindertageseinrichtungen und Schulen geschlossen, und eine Corona-Schutzverordnung schränkte das öffentliche Leben drastisch ein. Einzelhandel sowie Gaststätten und Restaurants mussten ihren Betrieb einstellen (Lockdown). Zusammenkünfte mit größeren Personengruppen wurden im öffentlichen Raum verboten. Soziale Öffentlichkeit und auch das politische Leben wurden auf ein Minimum reduziert. Bereits im März war zu erahnen, dass die Pandemie auf die bevorstehende Kommunalwahl in Nordrhein-Westfalen Mitte September 2020 dramatische Auswirkungen haben konnte.

Die folgende Arbeit greift die Konsequenzen der COVID-19-Pandemie auf die kommunalpolitischen Entscheidungsfindungsprozesse wie auch auf Kommunalwahlen auf. Dabei steht die Frage im Vordergrund, welche Sicherheitsmaßnahmen getroffenen wurden und wie diese Gegenmaßnahmen von den lokalen Akteuren und hier insbesondere von den LokalpolitikerInnen bewertet wurden.

## Methodisches Vorgehen und Forschungsfragen

Der Fokus der Untersuchung liegt auf Nordrhein-Westfalen. Die Kommunalwahl in dem bevölkerungsreichsten Bundesland, das mit 396 Kommunen zudem eine hohe Anzahl an größeren Städten vorweist, eignet sich besonders für eine Analyse. So fand hier der gesamte Prozess der Kommunalwahl unter den Restriktionen der Pandemie statt. Die Pandemie zeigte, dass im föderalen System unterschiedliche Herangehensweisen zur Krisenbewältigung getroffen wurden. Im Folgenden soll nicht darauf eingegangen werden, inwiefern die Maßnahmen in Nordrhein-Westfalen in strengerer oder abgemilderter Form hätten umgesetzt werden können. Die Untersuchung ist vielmehr eine Evaluation der politischen Prozesse und eine Bewertung der implementierten Strategien wie auch möglicher Alternativen.

Die empirischen Ergebnisse basieren dabei auf einer Online-Umfrage unter Ratsmitgliedern in ganz Nordrhein-Westfalen im Zeitraum Juli/August 2020. Dabei wurden Gemeinderäte aus allen nordrhein-westfälischen Großstädten über 100.000 EinwohnerInnen sowie eine gleiche Anzahl von Mittelstädten (zwischen 20.000 und 100.000) und Kleinstädten (unter 20.000) berücksichtigt. Die Kleingemeinden wurden nach einen Regionalproporz (Westfalen, Ruhrgebiert, Rheinland) mittels Zufallsprinzip ausgewählt. Insgesamt wurden 165 der 396 Gemeinden voll erfasst. Hier wurden – wenn möglich – alle Ratsmitglieder direkt angeschrieben. Insgesamt lag der Rücklauf bei etwa 1500 Ratsmitgliedern. Dabei ordneten sich 366 Ratsmitglieder der CDU, 429 der SPD, 172 Bündnis 90/Die Grünen, 89 der FDP und 50 der Linken zu. Bei allen anderen Parteien und Wählergruppen lag der Rücklauf unter 50 (insgesamt 147). Etwa 17 % gaben bei der Frage zur Parteizugehörigkeit keine Antwort. Damit entspricht der Rücklauf im Wesentlichen dem Parteienspektrum bei der NRW-Kommunalwahl 2014. Die CDU ist leicht unterrepräsentiert, SPD und Grüne leicht überrepräsentiert. Die AfD erreichte bei der Kommunalwahl 2014 nur 2,5 % der Stimmen und ist zudem leicht unterrepräsentiert. Sie ist daher mit den anderen kleineren Parteien und Listen in der parteispezifischen Analyse zur Kategorie „Kleinparteien“ zusammengefasst, da ansonsten bei Gruppen unter 50 Befragten die Anonymität nicht gewährleistet gewesen wäre. In dieser Kategorie dominieren die unabhängigen Wählerlisten.

Die Analyse rückt die lokalen politischen Entscheidungsprozesse und die Wahlen (Parteienregistrierung, Wahlkampf, Wahlumsetzung) in Zeiten der Pandemie in den Vordergrund. Hier konzentriert sich die Fragestellung auf das Verhalten der unterschiedlichen Parteien und das Rollenverständnis der Ratsmitglieder (Egner et al. 2013; Kersting 2016).

Dabei stellen sich unterschiedliche Forschungsfragen. Bewerten die VertreterInnen der Großparteien, die häufiger Regierungsverantwortung innehaben bzw. OberbürgermeisterInnen stellen, die restriktiven Beschränkungen der lokalpolitischen Entscheidungsprozesse weniger kritisch? Welche Einstellungsmuster zeigen sich bei den Mitgliedern von Kleinparteien und Freien Wählergruppierungen in Bezug auf die Registrierung bzw. die Vorbereitung auf die Wahl? Sehen diese den restringierten Wahlkampf unter Bedingungen einer Zwangsdigitalisierung eher problematisch? Treten Kleinparteien eher für eine Verschiebung der Wahl ein?

Weitere Fragestellungen, z. B., ob sich bei männlichen und weiblichen Ratsmitgliedern, bei unterschiedliche Altersgruppen, bei divergierenden Bildungsniveau der Ratsmitglieder oder auch in unterschiedlichen regionalen Kontexten (Klein‑, Mittel‑, Großstädte) signifikante Unterschiede zeigen, fließen kursorisch in die Analyse mit ein. Neben der quantitativen standardisierten Befragung hatten die Befragten die Möglichkeit, in einem offenen Feld Kommentare zu ergänzen, wovon viele Ratsmitglieder Gebrauch machten. An entsprechender Stelle fließen Ergebnisse der qualitativen Auswertung dieser Kommentare mit in die Auswertung ein. Abschließend soll auch die Frage aufgegriffen werden, ob sich aus den Ergebnissen Schlüsse und Handlungsempfehlungen für eine Post-Corona-Zeit ziehen lassen.

## Politische Entscheidungsfindungsprozesse in der Pandemie

Die rechtlichen Regelungen zur Bewältigung der Corona-Pandemie führten zur Schließung der Schulen sowie der Restaurants und des Einzelhandels am 13. März 2020 und wurden mit der Corona-Schutzverordnung verschärft (Land NRW 2020a). Das Versammlungsverbot galt für Gruppen von mehr als zwei Personen. Weiterführende Abstands- und Hygieneregeln betrafen insbesondere Risikogebiete. Vor dem Hintergrund mangelnder Infrastruktur und insbesondere eines Mangels an Masken und Diagnose-Tests wurde das öffentliche Leben weitgehend eingeschränkt. Erst Ende April wurde dies durch die Pflicht zum Tragen einer Mund-Nasenbedeckung im öffentlichen Raum erweitert (Paragraf 12 Abs. 1 Corona-Schutzverordnung).

Mitte April wurden Veranstaltungen, die der Aufrechterhaltung der öffentlichen Sicherheit und Ordnung oder der Daseinsvorsorge dienten, mit entsprechenden Vorkehrungen wieder erlaubt (Land NRW 2020b). Sinkende Infektionszahlen im Mai führten zu weiteren Lockerungen und begrenzten Öffnung von Schulen und Kitas. Im Sommer 2020 wurden in Nordrhein-Westfalen Veranstaltungen bis zu 300 Teilnehmern erlaubt, wenn entsprechende Hygieneschutzmaßnahmen, wie z. B. Mindestabstände, eingehalten wurden.

Auf der lokalen Ebene wurden bereits Anfang März Corona-Krisenstäbe eingesetzt, die zeitnahe und (sub-)lokale Lösungen entwickeln sollten. Das lokalpolitische Leben wurde weitgehend lahmgelegt. Demonstrative Beteiligung fand zunächst nicht statt und richtete sich dann vereinzelt gegen die Corona-Verordnungen und Maskenpflicht. Deliberative Bürgerbeteiligung in Planungsprozessen mit direkter Bürgerbeteiligung kam zum Stillstand (siehe zur Beteiligung im „invited space“ Kersting 2017). In der Mehrzahl übernahmen Hauptausschüsse die Entscheidungen, anstelle der Gemeinderäte (Jungvogel 2020). Aufgrund mangelnder Räume wurden zunächst weitergehende Lösungen, wie z. B. (analog zum Bundestag) Ratssitzungen mit halber Besetzung, nur begrenzt implementiert (z. B. in Rheine).

### Pandemie und Ratssitzungen

**Figure 1:**
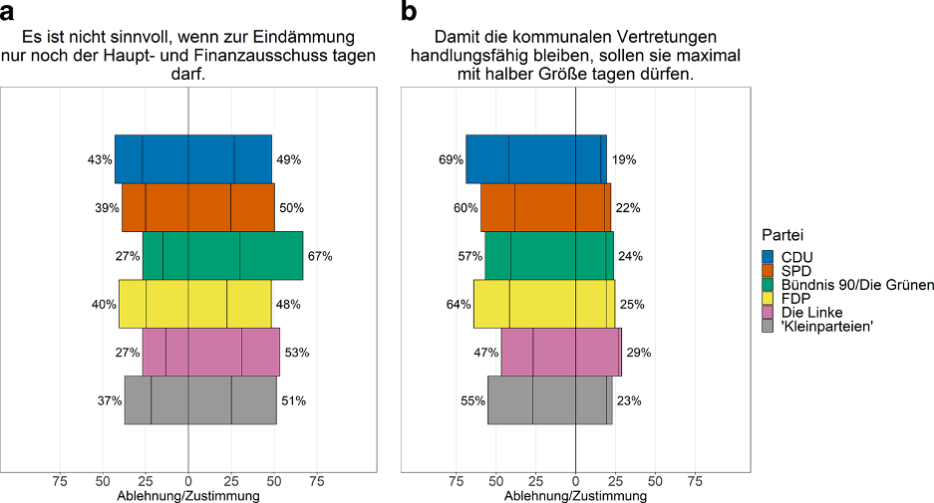

Bei den Umfragen im Juni/Juli 2020 bestand unter den Ratsmitgliedern eine große Unzufriedenheit über die Regelung während der ersten Welle der Pandemie (siehe Abb. 1). Die Aufwertung des Hauptausschusses wurde von den Parteien im Gemeinderat unterschiedlich bewertet (siehe Abb. 1). Durchschnittlich sah etwas mehr als ein Drittel der Ratsmitglieder dies als eine positive Lösung an. Mehr als die Hälfte (52 %) kritisierten diese Alternative. Hierbei zeigten sich Unterschiede zwischen den Parteien. Während CDU, FDP, SPD und die Kleinparteien dieser Regelung etwas positiver gegenüberstanden – von diesen Parteien waren zwischen 37 und 43 % damit zufrieden – lag die Zustimmung bei Grünen und Linken (je 27 %) deutlich niedriger. Auffällig ist, dass sich mehr als drei Viertel der LokalpolitikerInnen der Grünen (67 %) explizit gegen entsprechende Lösungen aussprachen.

Etwa ein Fünftel der LokalpolitikerInnen sahen Ratssitzungen mit halber Größe als (sehr) positiv an; fast zwei Drittel der Ratsmitglieder lehnten diese Lösung (sehr) stark ab. Die Skepsis überwiegt bei allen Parteien, am stärksten bei der CDU. Nur 19 % ihrer Ratsmitglieder sehen den Vorschlag positiv, aber 69 % negativ. Bei der Linken sind es immerhin 29 %, die diese Regelung positiv bewerten. Doch auch hier sind 47 % dagegen, und 24 % sehen es ambivalent. Schlüsselt man das Antwortverhalten nach Geschlecht auf, so zeigt sich, dass Frauen dieses Verfahren eher ablehnen als Männer.

Erst im Juli 2020 kam es in einigen Städten zur Revitalisierung der Gemeinderäte. Diese tagten in der Regel in größeren Sport- oder Messehallen. Hier bestand z. B. eine Maskenpflicht lediglich auf dem Weg in die Ratssitzung. Die Sitzungen waren durch lange Tagesordnungen geprägt, da viele Themen aufgearbeitet werden mussten und zudem die Sommerpause sowie die Kommunalwahl Mitte September 2020 vor der Tür standen.

Die Vielzahl der kritischen Kommentare der Ratsmitglieder über die offenen Felder zeigt, dass diesbezüglich die Kommunikation zwischen Politik und Verwaltung als stark defizitär betrachtet wurde. Ein Personalmangel in den Fachämtern erschwerte den direkten Kontakt. Aufgrund mangelnder Räumlichkeiten, die zudem oft als nichtadäquat genutzt betrachtet wurden, wurden viele Ratssitzungen, aber auch eine Vielzahl an Sitzungen von Kommissionen und Ausschüssen, nicht umgesetzt. Von den Ratsmitgliedern wurde kritisiert, dass Sitzungen durch die Verwaltungsspitze und die Fraktionsspitzen ohne die übrigen Ratsmitglieder nur eine unzureichende demokratische Meinungsbildung ermögliche. Der Ersatz der Ratssitzung durch den Hauptausschuss wurde insbesondere von den Kleinparteien kritisiert, da hier häufig fraktionslose Einzelvertreter im Hauptausschuss nicht stimmberechtigt sind.

Fraktionssitzungen wurden zum Teil als Telefonkonferenzen, aber häufiger als Videokonferenzen abgehalten, die nicht mehr als sechs TeilnehmerInnen mit ihrem Kamerabild erfassten und dabei den Informationsfluss und die Diskussion erschwerten. Grundsätzlich wurde die Qualität der Diskurse bei den Videokonferenzen bemängelt.

Bei den Rats- und Ausschusssitzungen, die lange Zeit gar nicht möglich waren, wurde kritisiert, dass Risikogruppen (Senioren, Personen mit Vorerkrankungen) von einer Teilnahme absehen. Zudem fehlten adäquate Kinderbetreuungsregelungen. Dies war auch deshalb problematisch, da eine Vielzahl der Ausschusssitzungen bereits am frühen Nachmittag beginnen.

Da insbesondere die Sitzungen der Ausschüsse ausfielen, wurde Kritik laut, dass die Handlungskompetenzen der Fraktionsspitzen und der höchsten WahlbeamtInnen, d. h. der BürgermeisterInnen, stark zunahmen. Eine Vielzahl der wichtigen Entscheidungen wurde mit hoher Dringlichkeit durch die Exekutive getroffen.

Auch die Kommunikation zwischen PolitikerInnen und BürgerInnen trat in dieser „Zeit der Exekutive“ in den Hintergrund. Aufgrund der Dauerpräsenz der BürgermeisterInnen sahen viele Befragte den Wahlkampf und die Wahlen als beeinträchtigt an. Dabei wurden die fehlende Integrität und Fairness aufgrund mangelhafter Chancengleichheit und deutlicher Vorteile der Exekutive und der AmtsinhaberInnen bemängelt.

### Pandemie und Parteienregistrierung zur Kommunalwahl

Die nordrheinwestfälischen Kommunalwahlen im September 2020 umfassten gemeinsame Gemeinderats- und Bürgermeisterwahlen (Holtkamp 2006; Holtmann et al. 2017). Für beide Wahlen begannen Anfang 2020, also etwa neun Monate vor der Kommunalwahl, die Vorbereitungsmaßnahmen, wie z. B. die KandidatInnen- und Listenaufstellung in den Parteien (Kersting 2017).

Vielfach hatten bei Ausbruch der Pandemie Mitte März die Listenparteitage noch nicht stattgefunden. Die Registrierung der Parteien, der Wählergruppen und der EinzelkandidatInnen bedarf einer Aufstellung von BewerberInnen in den entsprechenden Wahlbezirken. Im nordrheinwestfälischen Kommunalwahlgesetz wird zudem erwartet, dass sowohl für den Wahlbezirk als auch für die Reservelisten entsprechende Kandidaturen vorgehalten werden.

Nach dem nordrheinwestfälischen Kommunalwahlgesetz kann bis zum 59. Tag vor der Wahl ein Wahlvorschlag eingereicht und entsprechende Vertrauenspersonen benannt werden (Land NRW 2019, 2020c; Korte 2020). Dabei sind zudem Unterstützungsunterschriften notwendig: Es wird ein Promille der EinwohnerInnenzahl benötigt. Nur in besonderen Ausnahmefällen kann eine verspätete Unterzeichnung eingereicht werden. Die Reihenfolge der BewerberInnen auf den Reservelisten muss – auch wenn dies oft informell vorentschieden wurde – in einer Mitgliederversammlung bestimmt werden (Paragraf 17 Abs. 1 Kommunalwahlgesetz NRW vom 01.09.2019). Grundsätzlich sollte bis zum 40. Tag vor der Wahl die Zulassung des Wahlvorschlags entschieden werden. Daraus ergibt sich ein Beschwerderecht gegen die Entscheidung des Wahlausschusses (Paragraf 18 Art. 4 Kommunalwahlgesetz Nordrhein-Westfalen). Hierfür besteht für alle Wahlberechtigten das Recht, in die WählerInnenverzeichnisse Einblick zu nehmen. Diese Überprüfung kann zwischen dem 16. und 20. Tag vor dem Wahltermin erfolgen, um Einspruchsmöglichkeiten zu gewährleisten.

**Figure 2:**
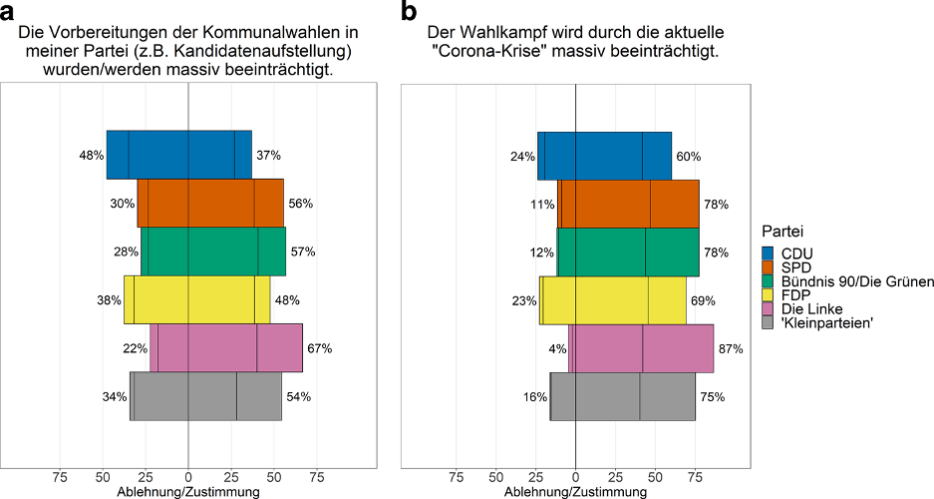

Diese Vorbereitungen der Kommunalwahl fielen letztendlich in die Hochphase der ersten Welle der COVID-19-Pandemie. 50 % der Ratsmitglieder sahen diese Vorbereitung als massiv beeinträchtigt an (vgl. Abb. 2). Von einer massiven Beeinträchtigung sprachen mehr als die Hälfte der Ratsmitglieder der Linken (67 %), von Bündnis 90/Die Grünen (57 %), der SPD (56 %) und der Kleinparteien (54 %). Knapp die Hälfte der FDP-Ratsmitglieder (48 %) stimmte der entsprechenden Aussage ebenfalls zu. Lediglich innerhalb der CDU sahen nur 37 % der befragten KommunalpolitikerInnen massive Beeinträchtigungen, wohingegen 48 % der Aussage nicht zustimmten.

Mit dem Lockdown im März 2020 gerieten das politische Leben und der Wahlkampf ins Stocken. Erst die Änderung der Kommunalwahlgesetze und die Erleichterungen führten zu einer Revitalisierung. Lange Zeit hielten sich Bedenken zum Wahltermin. So waren zum Teil neue Parteien und Wählerlisten, die Schwierigkeiten hatten, fristgerecht Unterstützungsunterschriften zu bekommen, zunächst zurückhaltend und erwarteten ein Urteil des Landesverfassungsgerichtshof und einen möglichen Aufschub der Wahl (Verfassungsgerichtshof NRW 2020). Ihnen waren aufgrund der Pandemie ohnehin die Hände gebunden. Ein Sammeln von Unterstützungsunterschriften war aufgrund der Angst vor Ansteckung bei längeren Gesprächen schwierig bis nahezu unmöglich.

Im Juni 2020 wurde das Kommunalwahlgesetz in Nordrhein-Westfalen verändert. Damit wurde die Frist zur Einreichung der Wahlvorschläge um 11 Tage verlängert. Gleichzeitig bekamen die Gemeinden acht Tage mehr Zeit zur Prüfung der Wahlvorschläge. Nur über diese Modifizierung der Regelung wurde die Registrierung von einigen Parteien und Wählergruppen erst ermöglicht. So konnten Wahlvorschläge bis zum 48. Tag vor der Wahl eingereicht werden (Paragraf 6 Gesetz zur Durchführung der Kommunalwahl 2020). Zudem wurde die Zahl der Unterstützung durch Unterschriften deutlich verringert (Paragraf 13 Gesetz zur Durchführung der Kommunalwahlen 2020).

Dennoch reichten die Maßnahmen für viele Initiativen und potenzielle Wählerlisten nicht aus. Der Zeitraum der Unterschriftensammlung wurde aufgrund der Verschiebung der Mitgliederversammlungen deutlich verkürzt. Es zeigten sich Fälle, wo Wahlvorschläge von unabhängigen Wählergruppen zurückgenommen wurden. Hiervon waren die Parteien und Wählergruppen, die bereits im Stadtrat vertreten sind, nicht betroffen. Doch auch sie waren verpflichtet, Mitgliederversammlungen abzuhalten, die vielfach zunächst verschoben, dann zum Teil als Online-Meetings geplant wurden und letztendlich aufgrund der Lockerung der Gesetzgebung oft doch als klassischer Präsenz-Parteitag stattfanden.

Das NRW-Ministerium für Heimat, Kommunales, Bau und Gleichstellung (MHKBG) forderte die Kommunen dazu auf, insbesondere neue Wählergruppen zu unterstützen. Große Probleme gab es dabei, entsprechende Räume für die Nominierungsveranstaltungen zu finden. Die hieraus resultierenden Kosten zur Einhaltung der Hygienebestimmung, d. h. für die besondere Bestuhlung, das Tragen von Mund-Nasenbedeckung und entsprechende Desinfektionsmittel, stellten viele Gruppierungen in einigen Städten dennoch vor enorme Herausforderungen.

Auch für die kommunalen Verwaltungen wurden Schwierigkeiten erwartet. Letztendlich stellte die Prüfung der Wahlvorschläge durch die kommunalen WahlleiterInnen aufgrund einer Aufstockung des Personals kein Problem dar. Es gab aber am Wahltag, an dem die Ratswahl, der erste Wahlgang der (Ober‑)BürgermeisterInnenwahl, die Wahl der Bezirksvertretungen sowie in einigen Kommunen die Landrats- und Kreistagswahlen und die Integrationsratswahlen stattfanden, zum Teil lange Wartezeiten.

In vielen Fällen haben die Listenparteitage nicht rechtzeitig stattgefunden. Die Zustimmungserklärung der Kandidaten entstand häufig unter enormen Zeitdruck. So fielen die Termine der Nominierungsveranstaltung zum Teil in die Urlaubszeit in Nordrhein-Westfalen.

Die Kommentare der Ratsmitglieder zeigen weiterhin, dass es während der Corona-Pandemie schwieriger war, KandidatInnen für die Besetzung der Wahlkreise anzusprechen und zu begeistern. Dies wurde nur zum Teil durch Befürchtungen sinkenden Handlungsspielraums aufgrund mangelnder finanzieller Kapazitäten begründet. Weiterhin waren Risikogruppen, insbesondere ältere Personen, schwerer zu kontaktieren. Die vom Innenministerium empfohlene kostenlose Überlassung von Räumen z. B. für Aufstellungsversammlungen wurde – so die Kommentare der Ratsmitglieder – nicht adäquat realisiert, und die Preise waren zu hoch. Die massive Beeinträchtigung der kleineren Parteien, die „analoge“ Unterstützungsunterschriften erbringen mussten (eine digitale Sammlung, Bearbeitung und Einreichung war nicht möglich), wurde stark kritisiert. Die hierzu genutzten Formulare wurden als „ineffiziente Schikane“ tituliert. Dies führte dazu, dass einige Wählergemeinschaften aufgrund mangelnder Chancengleichheit ihre Kandidatur bzw. Registrierung zurückzogen.

### Pandemie und Wahlkampf

Die Kommentare der Ratsmitglieder zu neuen, durch die Corona-Krise entstandenen Problemen beinhalteten insbesondere Befürchtungen stark sinkender kommunaler Einnahmen durch die starke Volatilität und Krisenanfälligkeit der kommunalen Gewerbesteuer. Hierdurch sah man den kommunalen Handlungsspielraum längerfristig eingeschränkt. Als unmittelbare Folge der Pandemie traten andere Politikfelder als mögliche Wahlkampfthemen zunächst in den Hintergrund.

Insbesondere neue BürgermeisterkandidatInnen hatten bereits im Januar und Februar 2020 mit der Wahlkampfplanung begonnen und erste Planungstreffen bzw. Wahlkampfveranstaltungen umgesetzt. Mit dem Lockdown im März 2020 kam dieser Wahlkampf weitgehend zum Erliegen. Mit der schrittweisen Öffnung des öffentlichen politischen Lebens wurden teilweise etablierte Wahlkampfinstrumente wieder möglich. Dennoch waren die klassischen Wahlkampfstände in den Innenstädten und Fußgängerzonen von den Schutzbestimmungen stark betroffen. LokalpolitikerInnen und insbesondere neue KandidatInnen konnten sich mit Mund-Naseschutz nicht entsprechend präsentieren. Die Wiedererkennung selbst der SpitzenpolitikerInnen wie der OberbürgermeisterInnen gelang nur bedingt. Neue unbekannte KandidatInnen hatten es besonders schwer sich darzustellen. So kam dem klassischen Plakat-Wahlkampf eine besondere Bedeutung zu. Das Verteilen von Werbeschriften wie auch die Hausbesuche waren aufgrund von Abstandsregelung und Ansteckungsgefahr eher problematisch.

Die Zwangsdigitalisierung, die sich im öffentlichen Leben, in Schulen und Hochschulen realisierte, zeigte sich auch im politischen Leben und dem Wahlkampf. Obwohl auf Bundesebene die Digitalisierung des Wahlkampfs in den letzten Jahren vorangetrieben worden war, lagen deutsche Bundes- und Landtagswahlkämpfe im Vergleich zu anderen Ländern, wie z. B. den USA, Frankreich oder Großbritannien, deutlich zurück. Kommunalwahlkämpfe hatten lange Zeit nur sporadische digitale Elemente. Mit der COVID-19-Pandemie wurde der digitale Wahlkampf insbesondere von den etablierten Parteien deutlich vorangetrieben. Dabei wurden verstärkt die sozialen Netzwerke Instagram und Facebook sowie in geringerem Maße Twitter genutzt. Eine Verknüpfung der Plakate über QR-Code erleichterte den Wechsel zwischen verschiedenen Kommunikationskanälen.

In der Umfrage im Juni/Juli 2020 sahen über alle Parteigrenzen hinweg fast drei Viertel der Ratsmitglieder in Nordrhein-Westfalen den Wahlkampf massiv durch die COVID-19-Pandemie beeinträchtigt (vgl. Abb. 2). Nur knapp ein Fünftel bewertete dies als weniger problematisch. Hier zeigen sich kaum Unterschiede zwischen den Parteien. Über drei Viertel der Linken- (87 %), SPD- und Grünen-Ratsmitglieder (je 78 %) sowie exakt 75 % aus den kleineren Parteien und Wählergruppen sahen diese Beeinträchtigung. Aber auch innerhalb der FDP und der CDU attestierten klare Mehrheiten der Ratsmitglieder (69 bzw. 60 %) massive Pandemie-Beeinträchtigungen im Wahlkampf. Nur je ein knappes Viertel der CDU- und FDP-Räte bewerteten dies als unproblematisch.

Weitere Analysen des Antwortverhaltens machen zudem deutlich, dass weibliche Ratsmitglieder eher eine massive Beeinträchtigung des Wahlkampfs wahrnahmen. Dies galt auch eher für die jüngeren KandidatInnen als für die älteren. Signifikant unterschiedliche Einschätzungen zeigten sich auch bei den EinwohnerInnen der Großstädte im Vergleich zu Klein- und Mittelstädten. In den letztgenannten sahen weniger Ratsmitglieder das Problem massiver Wahlkampfbeeinträchtigung. Dies mag damit zusammenhängen, dass Großveranstaltungen in Klein- und Mittelstädten weniger relevant sind und hier Veranstaltungen mit kleineren Gruppen auch in Zeiten der Pandemie eher realisiert werden konnten. In Großstädten wurden Podiumsdiskussionen und Großveranstaltung eher abgesagt.

In den offenen Fragen im Fragebogen konstatierten einige Ratsmitglieder zudem, dass aufgrund des Mangels an persönlichen Gesprächen die Kommunikation mit bildungsfernen Schichten weitestgehend ausgeblieben sei. Man beklagte, dass Telefongespräche oder E‑Mails dies nicht kompensieren könnten.

### Pandemie und der Wahlprozess

Bei der Festlegung des Wahltermins etwa ein Jahr vorab waren keine Anzeichen einer Pandemiekrise erkennbar. Die Pandemie führte zu 12 Verfahren des Verfassungsgerichtshofes Nordrhein-Westfalen; hier waren insbesondere unabhängige Personen, freie Wählergruppen und Kleinparteien, wie z. B. die Familien-Partei Deutschlands, Beschwerdeführer. Eine Verschiebung der Wahl auf den November 2020 oder in das Frühjahr 2021 wurde aufgrund der erwarteten Kontaktsperren im Winter negativ beschieden. Eine Verschiebung auf den Oktober war aufgrund der Überschneidung mit den Schulferien in Nordrhein-Westfalen problematisch und hätte zudem die aufgrund der Harmonisierung von BürgermeisterInnen- und Gemeinderatswahlen ohnehin lange Legislaturperiode zusätzlich verlängert.

**Figure 3:**
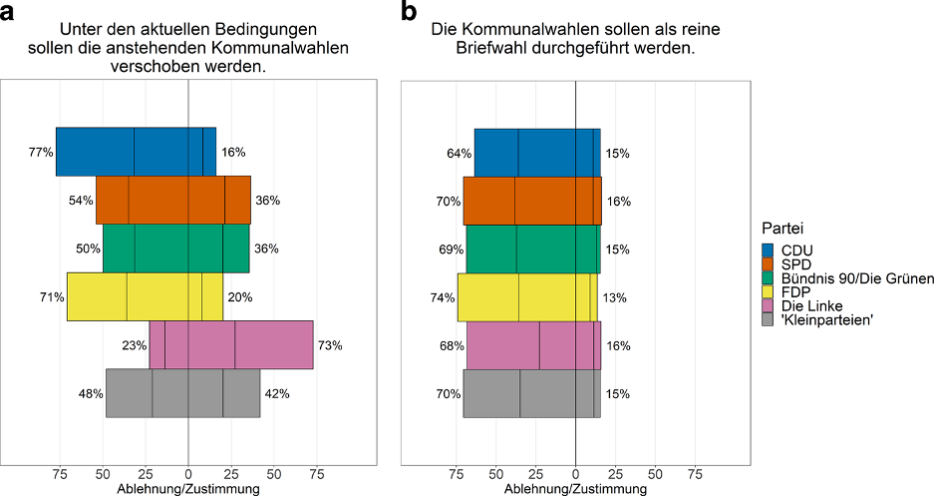

Die repräsentative Umfrage unter allen Ratsmitgliedern in Nordrhein-Westfalen zeigt, dass über alle Parteien hinweg weniger als ein Drittel für eine Verschiebung der Kommunalwahl war, während sich deutlich mehr als die Hälfte dagegen aussprachen (vgl. Abb. 3). Hier sind deutliche Unterschiede zwischen den Parteien erkennbar. Insbesondere bei der Linkspartei (73 %) hätte man sich eine Verschiebung der Wahl gewünscht. Bei allen anderen Parteien war jeweils eine relative Mehrheit gegen die Verschiebung. Am stärksten gegen eine Verschiebung der Wahl sprachen sich die VertreterInnen von CDU (77 %) und FDP (71 %) aus. Bei SPD und Grünen war jeweils ein gutes Drittel für eine Verschiebung, etwa die Hälfte dagegen. Die Ratsmitglieder der Kleinparteien waren in dieser Frage am ambivalentesten (42 % dafür, 48 % dagegen).

Untersucht man das Antwortverhalten hinsichtlich weiterer Kriterien, zeigen sich signifikante Unterschiede. Ratsmitglieder mit höherem und universitärem Bildungsabschluss sprachen sich eher gegen eine Verschiebung der Wahl aus. Gleiches gilt für die Ratsmitglieder in den Klein- und Mittelstädten.

Mit den Änderungen des Kommunalwahlgesetzes von 2020 wurde es ermöglicht, die Stimmbezirke von 2500 Wahlberechtigten auf 5000 Wahlberechtigte aufzustocken. Hierüber sollte ein Mangel an Wahllokalen vermieden werden. Aufgrund dieser Regelung kam es trotz der hohen Zahl an Vorabwählern (Briefwählern) in vielen Städten und Wahllokalen zu langen Schlangen und Wartezeiten.

Aufgrund der Abstandsregelungen und verschiedener Sicherheitsbedenken konnten unterschiedliche, lange Zeit etablierte Wahllokale nicht benutzt werden. So standen vielfach kleinräumige Kindergarteneinrichtungen nicht zur Verfügung. Von der Nutzung von Altersheimen und Pflegeeinrichtungen, die Risikogruppen beherbergen, wurde abgeraten. Die gesamte Planung wurde eng mit den Corona-Krisenstäben und den örtlichen Gesundheitsämtern entwickelt. Das Vermummungsverbot wurde aufgehoben und WahlhelferInnen und Wahlvorständen wurde es erlaubt und angeraten, Mund- und Nasenschutz zu tragen. Die Risikogruppen (über 65-Jährige) wurden nicht als WahlhelferInnen verpflichtet. Die Zahl der WahlhelferInnen wurde erweitert, häufig durch das Heranziehen von MitarbeiterInnen der Kommunalverwaltungen.

Die Attraktivität der Briefwahl nimmt seit Jahrzehnten zu (Kersting 2019). Bei der Bundestagswahl lag der Anteil bei etwa einem Drittel der WählerInnen. Auch die NRW-Kommunalwahl im September 2020 zeigte einen starken Zuwachs der BriefwählerInnen (etwa zwei Drittel der WählerInnen [Landeswahlleiter NRW 2020]). Die Stichwahl der Kommunalwahl in Bayern im März 2020 fiel in die erste Welle der Corona-Pandemie. Dort entschied man sich dafür, die gesamte Stichwahl ausschließlich als Briefwahl abzuwickeln, d. h. es gab keine Präsenzwahl. Diese Option einer reinen Briefwahl hätte grundsätzlich auch für NRW bestanden.

Auch zu dieser Möglichkeit wurden die Ratsmitglieder zwei bis drei Monate vor der Kommunalwahl in Nordrhein-Westfalen befragt (s. Abb. 3). Zu diesem Zeitpunkt wurden bereits die vorab restriktiveren Maßnahmen abgemildert. Nur etwa jedes siebte Ratsmitglied wollte die Kommunalwahl als reine Briefwahl durchführen. Die Zustimmung zwischen den Parteien schwankt mit 13–16 % nur gering. Mehr als zwei Drittel sprachen sich hingegen gegen diese Alternative aus. Die stärksten Widerstände gegen die reine Briefwahl zeigten sich bei der FDP (74 %), die geringsten bei der CDU (immerhin auch noch 64 %). Weitere Analysen zeigen zudem signifikante Unterschiede für Kleinstädte, wo Briefwahl auf eine höhere Akzeptanz stößt.

## Schlussfolgerungen für die Post-Corona-Lokalpolitik

Die erste Welle der COVID-19-Pandemie im Frühjahr und Frühsommer 2020 hatte nicht nur Auswirkungen auf das gesellschaftliche Leben allgemein, sondern im Speziellen auch auf politische Entscheidungsprozesse und Wahlen. Die hieraus resultierende Zwangsdigitalisierung zeigte Defizite beim Ausbau von Glasfaser-Kabeln und Infrastruktur wie auch Mängel an adäquaten Software-Lösungen (Kersting und Graubner 2020). Die Corona-Krise zeigte somit auch, was Digitalisierung kann, was sie nicht kann und was sie noch nicht kann.

Die Kontaktbeschränkungen, das Verbot von Gruppenveranstaltungen und der Lockdown betrafen das politische System auch auf der lokalen Ebene. Die häufige Kritik und die Diskussion über die Einschränkung des Demonstrationsrechts und anderer Formen der demonstrativen Demokratie übersehen leicht, dass vielfältige Instrumente der deliberativen Partizipation (Offene Foren, Stakeholder-Konferenzen, Bürgerräte) des „invited space“ und „invented space“ zur informellen Bürgerbeteiligung insbesondere auf kommunaler Ebene gar nicht stattfinden konnten. Aber auch die Beteiligung und Entscheidungsprozesse der repräsentativen Demokratie wurden stark eingeschränkt. So mussten die Ratssitzungen in der gewohnten Form ausfallen und wurden durch Sitzungen des Hauptausschusses ersetzt. Einige Kommunen experimentierten am Ende der ersten Welle der Pandemie mit Gemeinderäten, die in halber Besetzung antraten bzw. später mit Hygiene-Regelungen abgehalten wurden. Viele Ausschusssitzungen und Kommissionen fielen vollständig aus. Dies führte zu einer problematischen Dominanz der Exekutive und zudem zu einer mangelnden Inklusion, vor allem kleinerer Wählergruppen und Parteien. Diese Defizite wurden über alle Parteigrenzen hinweg von allen Ratsmitgliedern in NRW mehrheitlich stark kritisiert. Bei der Registrierung zu den bevorstehenden Kommunalwahlen im September 2020 wurden kleinere Parteien und neue KandidatInnen gegenüber den etablierten Parteien und den AmtsinhaberInnen benachteiligt. Vielfach wurde deshalb ein Verschieben der Kommunalwahl erwartet, was aber eher aus organisatorischen Gründen vom Landesverfassungsgerichtshof abgelehnt wurde, da die pandemische Situation unklar war und es die Legislaturperiode deutlich über das Normalmaß verlängert hätte. Die relevanten Fristen zur Registrierung und die administrativen Anforderungen wurden vom Land zwar erleichtert, dennoch führte die Corona-Schutzverordnung sowie auch mangelnde Infrastruktur und Räumlichkeiten dazu, dass viele Parteien behindert wurden und unabhängige Wählerlisten ihre Registrierung zurückzogen. Von den Ratsmitgliedern der etablierten Parteien wurde ein Verschieben der Wahl abgelehnt. Alle Parteien lehnten auch eine reine Briefwahl ab.

Im Wahlkampf kam es zudem bei neuen Parteien und KandidatInnen zu erheblichen Einschränkungen. Hier wurde zusätzlich der Mangel an digitalen Publikationsmitteln mit entsprechenden elektronischen Signaturmöglichkeiten kritisiert. Auch der Wahlkampf selbst war für kleinere Parteien und unabhängige neue Kandidaten eher problematisch. Zentrale Instrumente wie der Straßenwahlkampf (hinter der Maske), Podiumsveranstaltung und Hausbesuche mussten weitgehend reduziert werden. Letztere fanden eher in kleineren Gemeinden ein stärkeres Interesse. Bislang ist in Deutschland Digitalisierung der Wahlkämpfe sowohl auf nationaler, auf regionaler, aber insbesondere auf lokaler Ebene wenig fortgeschritten. Diese Digitalisierung bekam insbesondere bei den Großparteien, welche über die notwendigen finanziellen Mittel, Hilfen durch Bundes- und Landespartei sowie eine Unterstützung durch eigene Parteimitglieder mit entsprechenden Qualifikationen verfügten, einen enormen Schub. Hier wurden zum Teil erstmalig in breiter Front soziale Medien, insbesondere Facebook und Instagram, genutzt. Grundsätzlich war innerhalb der Ratsmitglieder die Kritik an der Umsetzung des Wahlkampfs aufgrund der massiven Einschränkungen durch die Pandemie überdeutlich. Alternative Instrumente als Online-Wahlhilfen (Kommunalwahlcheck, Wahl-Kompass, Lokal-O-Mat u. a.) standen zumeist nur in einigen wenigen Großstädten zur Verfügung.

Insofern stellt sich auch hier die abschließende Frage, ob die Kommunen stärker – über die Ratsinformationssysteme hinaus – digitale Verfahren für kommunale Entscheidungsprozesse der Räte und Ausschüsse (elektronische Parlamente) implementieren sollten. Auch digitale Beteiligungsinstrumente z. B. zur Registrierung von Wählergruppen und Parteien und auch entsprechende politische Informationsplattformen für die Bürger müssen in höherem Maße im Rahmen der erweiterten kommunalen Daseinsvorsorge vorgehalten werden.
